# Functional Characterization of the Lysosomal Peptide/Histidine Transporter PHT1 (*SLC15A4*) by Solid Supported Membrane Electrophysiology (SSME)

**DOI:** 10.3390/biom14070771

**Published:** 2024-06-28

**Authors:** Jonai Pujol-Giménez, Sven P. Baumann, Tin Manh Ho, Bartlomiej Augustynek, Matthias A. Hediger

**Affiliations:** 1 Department of Nephrology and Hypertension, Inselspital, University of Bern, Kinderklinik, Freiburgstrasse 15, 3010 Bern, Switzerlandmanh.ho@unibe.ch (T.M.H.); bartlomiej.augustynek@unibe.ch (B.A.); matthias.hediger@unibe.ch (M.A.H.); 2 Department of Biomedical Research, Inselspital, University of Bern, Kinderklinik, Freiburgstrasse 15, 3010 Bern, Switzerland; 3 Graduate School for Cellular and Biomedical Sciences, University of Bern, 3012 Bern, Switzerland

**Keywords:** SLC15 family, *SLC15A4*, PHT1, solid supported membrane electrophysiology (SSME), functional characterization, substrate selectivity, pH dependence, molecular docking

## Abstract

The peptide/histidine transporter PHT1 (*SLC15A4*) is expressed in the lysosomal membranes of immune cells where it plays an important role in metabolic and inflammatory signaling. PHT1 is an H^+^-coupled/histidine symporter that can transport a wide range of oligopeptides, including a variety of bacterial-derived peptides. Moreover, it enables the scaffolding of various metabolic signaling molecules and interacts with key regulatory elements of the immune response. Not surprisingly, PHT1 has been implicated in the pathogenesis of autoimmune diseases such as systemic lupus erythematosus (SLE). Unfortunately, the pharmacological development of PHT1 modulators has been hampered by the lack of suitable transport assays. To address this shortcoming, a novel transport assay based on solid-supported membrane-based electrophysiology (SSME) is presented. Key findings of the present SSME studies include the first recordings of electrophysiological properties, a pH dependence analysis, an assessment of PHT1 substrate selectivity, as well as the transport kinetics of the identified substrates. In contrast to previous work, PHT1 is studied in its native lysosomal environment. Moreover, observed substrate selectivity is validated by molecular docking. Overall, this new SSME-based assay is expected to contribute to unlocking the pharmacological potential of PHT1 and to deepen the understanding of its functional properties.

## 1. Introduction

Among the large number of human genes dedicated to membrane transport processes, up to half encode members of the solute carrier (SLC) supergroup of membrane transport proteins. This supergroup also includes the major facilitator superfamily, to which the SLC15 proton-coupled oligopeptide transporters (POTs) belong (reviewed in [1]). The transporters of the SLC15 family have 12 transmembrane-spanning regions with both amino and carboxy termini facing the intracellular side and pseudosymmetry between the structural motifs [2]. To date, five different membrane transporters encoded by the SLC15 family have been identified. The oligopeptide transporters PepT1 (*SLC15A1*) and PepT2 (*SLC15A2*) are mainly expressed in the apical membranes of the small intestine and kidney proximal tubules, respectively, and their physiological roles have been well characterized [3]. In contrast, the peptide/histidine transporters PHT2 (*SLC15A3*) and PHT1 (*SLC15A4*), are expressed in the endosomal and lysosomal membranes of immune cells, where they play essential roles in metabolic and inflammatory signaling events [4]. The recently discovered member of this family, *SLC15A5*, was identified by bioinformatic approaches and its expression profile and functional properties are still unclear [5]. 

Members of the SLC15 family are known for their substrate promiscuity, as they can transport a wide range of oligopeptides and a variety of peptide-like drugs. PepT1 and PepT2 substrates include di- and tripeptides, beta-lactam antibiotics and angiotensin converting enzyme (ACE) inhibitors, among many others [6]. Interestingly, in addition to di- and tripeptides, PHT2 and PHT1 have been shown to transport free AAs, such as histidine [6]. Concerning PHT1, it has been shown to transport carnosine, valacyclovir, and bacterial-derived peptides such as muramyl dipeptide (MDP) or L-Ala-γ-D-Glu-meso-diaminopimelic acid (Tri-DAP), among other nucleotide-binding oligomerization domain-like receptor (NOD) ligands [1,2,4,7,8,9]). Unfortunately, experimental validation and detailed kinetic analysis for these substrates is still incomplete and has been hampered by the localization of PHT1 in the endosomal–lysosomal organelles. Various expression systems have been used to generate plasma membrane expression for functional analysis. However, there are significant inconsistencies in the published work with respect to substrate selectivity, transport kinetics of the identified substrates, and pH-dependence. For example, the first functional studies with rat PHT1 using *Xenopus laevis* oocytes as an expression system reported histidine transport (K_M_ = 17 µM) with maximum activity at pH 5.5 [7]. Another study using human PHT1 transiently transfected COS-7 cells confirmed the transport of histidine but not glycylsarcosine (Gly-Sar), with maximum activity observed at pH 5.0 [10]. In contrast, in a subsequent work using human PHT1 stably transfected COS-7 cells, the same group reported the pH-independent transport of histidine. Moreover, this time Gly-Sar appeared to be a good substrate for PHT1-mediated efflux, and surprisingly, transport kinetics analysis for histidine revealed a K_M_ in the low millimolar range (K_M_ = 0.16 mM) [11]. In a more recent study using a mutant variant of human PHT1 in stably transfected MDCK cells, histidine and Gly-Sar transport was confirmed. Interestingly, while the histidine transport kinetics (K_M_ = 16 µM) were in line with initial studies, in contrast to previous pH-dependence reports, maximum transport activity was reached at pH 6.5 and decreased at more acidic extracellular pH conditions [3,8]. In another recent work, using purified chicken PHT1, no protein stabilization (i.e., binding) was observed in the presence of histidine at a concentration of 5 mM, whereas protein stabilization was observed under the same conditions in the presence of a variety of di- and tripeptides containing lysine and arginine, but not histidine or glycine [2].

PHT1 (*SLC15A4*) was cloned from a rat brain cDNA library [7], while the human homologue was cloned from an intestinal cDNA library and Caco-2 cells [12]. The human *SLC15A4* gene encodes a 577-amino acid membrane protein whose expression has been found primarily in the brain, intestine, some tumor cells, and a variety of immune cells, including monocytes/macrophages and dendritic cells [3]. PHT1 has been shown by immunofluorescence to be localized in lysosomes and late endosomes [13], where it plays a pivotal role in lysosomal activity by controlling the levels of certain amino acids (AAs) and oligopeptides and/or by scaffolding various signaling molecules involved in different metabolic and inflammatory events [4]. Due to the pH buffering ability of histidine, H^+^-coupled transport of this amino acid by PHT1 has been proposed to contribute to the regulation of lysosomal acidic levels, which is key for the activity of lysosomal enzymes, such as cathepsins, or the integrity of transport proteins, such as the V-ATPase. In this context, dysregulation of lysosomal histidine homeostasis due to PHT1 dysfunction has been associated with impaired Toll-like receptors (TLRs) mediating type I interferon (IFN-I) production [13,14], and also, with a failure of the IFN regulatory factor 7 (IRF7)-IFN-I regulatory circuit due to disruption of the mammalian target of rapamycin (mTOR) pathway [15]. In addition, PHT1-mediated transport of bacterial oligopeptides, such as MDP or Tri-DAP, triggers the activation of nuclear factor-kappa-B (NF-ΚƁ), which modulates the NOD-dependent immune response [3]. In terms of scaffolding activity, it has been shown that PHT1 interacts with Ragulator components such as LAMTOR1 and LAMTOR2, suggesting that it may be a component of the mTORC1 supercomplex [16]. In addition, PHT1 was demonstrated to interact with the “TLR adaptor interacting with SLC15A4 on the lysosome” (TASL), which modulates TLR7, TLR8, and TLR9 signaling and mediates IFN-I production by recruiting the transcription factor IRF5 [17]. Taken together, these findings demonstrate that PHT1 (*SLC15A4*) plays a key role in the immune response, so it is not surprising that several studies have implicated PHT1 in the development of diseases associated with immune dysfunction, such as systemic lupus erythematosus (SLE) [18,19,20,21,22] and inflammatory bowel disease (IBD) [19]. In addition, PHT1 has been implicated in other human diseases such as type 2 diabetes [23], combined pituitary hormone deficiency [24], and lung adenocarcinoma [25].

As just described, PTH1 transport and scaffolding functions may affect the lysosome environment [4,26], enable NOD and TLR signaling [3,13,14], and modulate mTOR activity in human immune cells [4,15]. Furthermore, it appears to play a pivotal role in the pathogenesis of SLE [18,19,20,21,22]. Therefore, molecules capable of modulating PHT1 functions have great potential for therapeutic interventions aimed at modulating the immune response, such as required for SLE treatment. Despite the obvious therapeutic interest of PHT1, its pharmacological development has unfortunately been hampered by the lack of appropriate functional assays and limited structural information. With the aim to provide appropriate tools for the pharmacological development of PHT1, and address the inconsistencies surrounding the current understanding of its functional properties, the present work presents a new transport assay based on solid supported membrane electrophysiology (SSME) [27]. Using this novel SSME method, a characterization of some interesting functional properties of PHT1 is presented, including substrate selectivity among a variety of AAs and peptides, pH-dependence, and transport kinetics of the identified substrates L-histidine, L-arginine, L-lysine, His-Leu, and Leu-Leu. Notably, in contrast to previous functional studies developed for PHT1 [7,8,10,11], this work was carried out with isolated lysosomal membranes, allowing the protein to be studied in its native environment.

## 2. Materials and Methods

All chemicals and reagents were purchased from Merck (Schweiz), Zug, Switzerland unless otherwise stated.

### 2.1. Cell Culture

HEK293 cells were obtained from American Type Culture Collection (ATCC) (Manassas, VA, USA) cultured in Dulbecco’s modified Eagle medium (DMEM) supplemented with 10% foetal bovine serum (FBS), 10 mM HEPES, 1 mM sodium pyruvate, 100 μM minimal essential medium (MEM) non-essential AAs, and 100 units/mL penicillin/streptomycin and maintained under standard cell culture conditions (37 °C; 5% CO_2_). To generate a cell line stably overexpressing (OE) human *SLC15A4* (PHT1), HEK293 cells were transfected with the DNA construct encoding the PHT1-GFP C-term fusion protein, using Lipofectamine 2000 (Thermo Fisher Scientific, Basel, Switzerland) transfection reagent according to the manufacturer’s protocol. The GFP-PTH1 OE cell line was obtained by a selection strategy based on resistance to G418 antibiotic (Thermo Fisher Scientific, Basel, Switzerland). The culture media used to maintain HEK293 GFP-PHT1 OE cell line were also supplemented with 500 μg/mL G418. 

### 2.2. Fluorescence Imaging

HEK293 cells were plated on coverslips (12 mm diameter) (Carl Roth Ag, Diessenhofen, Switzerland) in 12-well plates (Corning by Thermo Fischer Scientific, Basel, Switzerland) at a density of 100,000 cells per well. After a 24-h incubation, the cells were exposed to media containing Lysotracker (Thermo Fischer Scientific, Basel, Switzerland). The Lysotracker media were prepared by diluting the stock solution (1 mM) to a working concentration of 75 nM. Subsequently, the culture media (Thermo Fischer Scientific, Basel, Switzerland) were replaced with prewarmed (37 °C) Lysotracker-containing media and the cells were incubated for 2 h under standard growth conditions. The cells were then fixed with 4% paraformaldehyde (PFA) (Electron Microscopy Sciences, Hatfield, PA, USA) at 4 °C for 10 min. The coverslips were then rinsed thrice with phosphate-buffered saline (PBS) (Thermo Fisher Scientific, Basel, Switzerland) and incubated in PBS for 1 h. DNA staining was performed using Hoechst 33342 (10 mg/mL stock solution) (Thermo Fisher Scientific, Basel, Switzerland) at a dilution of 1:5000 for 20 min at room temperature (RT). Subsequently, the coverslips were washed twice with PBS for 40 min. Finally, the coverslips were mounted with ProLong™ Diamond Antifade Mountant (Thermo Fisher Scientific, Basel, Switzerland) and allowed to cure overnight at 4 °C. Imaging was conducted using a Leica SP8 confocal microscope (Leica Microsystems AG, Balgach, Switzerland) with a 63× objective, oil immersion, and digital zoom from 3× to 5× (*n* = 1.1513 at 23 °C). Image deconvolution was performed using Huygens Professional version 24.04 (Scientific Volume Imaging, Hilversum, The Netherlands), licensed by the Microscopy Imaging Center at the University of Bern.

### 2.3. Isolation of Lysosomal Fraction

HEK293 GFP-PHT1 OE cells or HEK293 wild-type cells were washed with PBS, trypsinized, and centrifuged at 500× *g* for 5 min to produce cell pellets of approximately 2 g. Cell pellets were then resuspended in 5 mL sucrose homogenization buffer (250 mM sucrose, 20 mM HEPES, and cOmpleteTM protease inhibitor cocktail; pH 7.4 adjusted with NMDG) and homogenized by sonication (3 × 10 sonication pulses, 0.5 s, 20% potency). Homogenates were centrifuged at 500× *g* for 5 min at 4 °C to discard cell debris (P1). The resulting supernatant (S1) was centrifuged at 6800× *g* for 10 min at 4 °C. The pellet (P2) containing the mitochondrial fraction was discarded and the remaining supernatant (S2) was centrifuged again at 20,000× *g* for 30 min to generate a pellet (P3) containing the lysosomal fraction. S3 was discarded and P3 was resuspended in 5 mL ice-cold sucrose homogenization buffer and centrifuged at 20,000× *g* at 4 °C for 15 min. The supernatant (S4) was discarded and the remaining pellet (P4) containing a clean lysosomal fraction, was resuspended in 10 mM citric acid buffer at pH 6.0 supplemented with the cOmpleteTM protease inhibitor cocktail and stored until use at −80 °C. 

### 2.4. Western Blot Analyses

Protein concentration was determined by Bradford colorimetric assay (Bio-Rad Europe GmbH, Basel, Switzerland). Equal amounts of lysosomal membrane preparations samples (10 µg) were resolved in 8% and transferred onto PVDF membranes. Immunodetection was achieved using the following antibodies: mouse monoclonal anti-GFP (SC-9996, Santa Cruz Biotechnology Inc., Heidelberg, Germany) (1/1000 dilution), rabbit polyclonal anti-PHT1 (ab64429, Abcam, Cambridge, UK) (1/200 dilution), mouse monoclonal anti-CD71 (#13-6800, Invitrogen, Carlsbad, CA, USA)) (1/200 dilution), rabbit polyclonal anti-Na^+^/K^+^-ATPase (stj94337, Lucerna-Chem Ag, Luzern, Switzerland) (1/200 dilution), mouse monoclonal anti-p53 (#2524s, Cell Signaling Technology, Danvers, MA, USA) (1/200 dilution), mouse monoclonal anti-LAMP1 (ab25630, Abcam, Cambridge, UK) (1/1000 dilution), rabbit anti-GPP130 (epr13439, Abcam, Cambridge, UK) (1/200 dilution), mouse monoclonal anti β-actin (SC-47778, Santa Cruz Biotechnology Inc., Heidelberg, Germany) (1/2000 dilution), primary antibodies, goat anti-mouse IgG (#1721011, Bio-Rad Europe GmbH, Basel, Switzerland) (1/3000 dilution), and goat anti-rabbit IgG (w4011, Promega AG, Dübendorf, Switzerland) (1/10,000 dilution) secondary antibodies. Antibody recognition signal was revealed by enhanced chemiluminescence detection reagent and blot images were acquired with the ChemiDoc Imaging System (Bio- Rad Europe GmbH, Basel, Switzerland). 

### 2.5. Microscale Thermophoresis (MST)

MST measurements were performed with the Monolith NT.115 instrument (NanoTemper Technologies GmbH, Munich, Germany). Membrane preparation of HEK293 cells overexpressing PHT1-GFP were obtained as described elsewhere [28]. Equal amounts of the membrane preparation were loaded into Monolith capillaries (NanoTemper Technologies GmbH, Munich, Germany; MO-K022) together with the indicated concentrations of L-histidine; both dissolved in 10 mM citric acid buffer at pH 5.0. To measure the MST, a temperature gradient was generated by infrared (IR) laser (80% of IR laser power) and fluorescence (60% of Blue LED power) was recorded continuously for 35 s. Data were collected with MO. Control v2 and analyzed with MO. Affinity v2.3 software (NanoTemper Technologies GmbH, Munich, Germany). 

### 2.6. Solid Supported Membrane-Based Electrophysiology (SSME)

The initial protein concentration of the lysosomal fraction preparations used for the SSME experiments was between 1.5 and 3.5 mg/mL. Prior to sensor preparation, to load the same amount of total protein, the protein concentration was adjusted in all experimental groups and then the samples were diluted 1:10 in 10 mM citric acid buffer at pH 5.0. SSME experiments were conducted using the SURFE^2^R N1 device (Nanion Technologies GmbH, Munich, Germany) and 3 mm diameter gold sensors were prepared according to the standard protocol recommended by the manufacturer. Current traces were recorded using the SURFE^2^R N1 Control V1 1.6.0.1 software (Nanion Technologies GmbH, Munich, Germany). Currents were recorded for the entire duration of the solution exchange protocol and amplified with a current amplifier set to a gain of 10^9^ V/A. All current traces shown in the same graph were recorded on the same sensor. For data analysis, magnitude of the peak of the current observed during the perfusion of the activating solution (Peak_on_) were used. To average data obtained from different sensors, the recorded Peak_on_ currents were normalized to the control condition, which was the Peak_on_ current magnitude recorded for L-histidine 10 mM in citric acid buffer at pH 5.0. 

### 2.7. Molecular Docking

Transporter coordinate files, 8P6A and Q8N697, were downloaded from the PDB (http://www.rcsb.org/) and AlphaFold (https://alphafold.com/) databases, respectively. The ligand coordinate files were downloaded from Pubchem (https://pubchem.ncbi.nlm.nih.gov/). The Pubchem IDs are as follows: 6274 (L-histidine), 5962 (L-lysine), 5950 (L-alanine), 6106 (L-Leucine), 6322 (L-Arginine), 6137 (L-Methionine), 9313 (Gly-Sar), 7687 (Leu-Leu), 189008 (His-Leu), 11163 (Gly-Gly), 11161 (Gly-Gly-Gly), 44093 (Captopril), and 5362119 (Lisinopril). The individual docking experiments were performed using the AutoDock Vina tool of the UCSF Chimera software version 1.17.3. Prior to molecular docking, the energy of the protein structures was minimized using standard parameters. A docking box containing the putative PHT1 substrate binding site was defined using the following parameters: 8P6A (center: 120, 120, 121; size: 13, 23, 20) and Q8N697 (center: 0.6, 0.13, 0.8: size: 25, 26, 16). The standard AutoDock Vina protocol was run, and the best scores were annotated. The pdb2pqr tool was used for the adjustment of the protonation state of the protein structures to pH 5.0 [29]. The H-bonds and residues involved were identified by visual inspection of the docking results showing the best scores.

### 2.8. Molecular Dynamics (MD) Simulation

The PDBQT files generated by molecular docking were used as the starting point for the MD simulations. PDB files containing the protein structure and the corresponding best-scoring ligand docking conformations were generated. The optimal positioning of the protein on the lipid membrane was adjusted using the PPM 2.0 web server [30]. The system required for the MD simulations was generated using the CHARM-GUI platform [31]. Proteins were embedded in rectangular membranes consisting of palmitoyl–oleoyl–phosphatidyl–choline (POPC) and 150 mM NaCl ions were included in the simulation system. Simulations were performed with the CHARMM36m force field at a temperature of 303.15 K on UBELIX (https://www.id.unibe.ch/hpc), the High-Performance Computing (HPC)^3^ cluster at the University of Bern, using AMBER 20 [32,33]. Initial system equilibration and MD simulations were performed using the module pmemd.cuda of AMBER20. The simulation covered a time spam of 150 ns with calculation steps set to 2 fs. The generated 10 ns simulation segments were concatenated with the cpptraj.cuda AMBER20 module. The final simulations were saved with a resolution of 20 frames per ns. For the analysis of the simulations, VMD and Python module MDA analysis were used. First, major errors were ruled out by visual inspection using VMD. Next, a region of 5 Å around the docking site was selected to assess the stability of the ligand docking conformation along the simulation using the MDA analysis sphzone command.

### 2.9. Statistics

Comparison of means between experimental groups was performed using the unpaired Student’s *t*-test or the Mann–Whitney U test. The test was selected for each case according to the fit of the data to the normal distribution as assessed by the Shapiro–Wilk test. All statistical tests were performed with IBM SPSS Statistics 20 software. The significance level was set at *p* < 0.05.

## 3. Results

**Figure 1:**
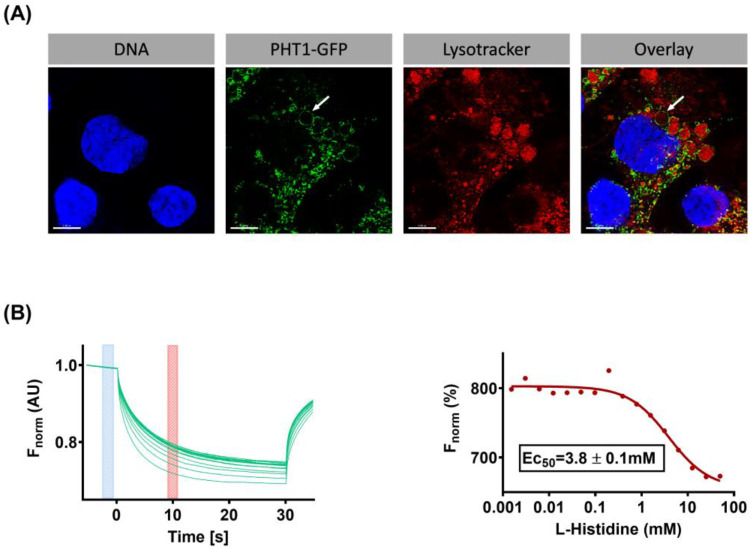
Fluorescence imaging of the HEK293 PHT1-GFP OE cell line and assessment of PHT1-GFP functional activity by microscale thermophoresis (MST). (**A**) Representative image showing the fluorescence signal emitted at different wavelengths by the fluorophores Hoechst (DNA staining; blue colour), GFP (PHT1-GFP overexpression; green colour), and Lysotracker (acidic compartment staining; red colour). Overlay of the different fluorescence signals (**right panel**). An illustrative example of the location of PHT1-GFP in lysosomal membranes is indicated by an arrow. The depicted scale bars correspond to 5 µm. (**B**) (**Left panel**) MST traces showing fluorescence measurement upon IR stimulation of capillaries loaded with a membrane preparation isolated from HEK293PHT1-GFP OE cells and different concentrations of L-histidine (0.0015–50 mM). Time points selected to calculate the change in fluorescence (ΔF) upon IR stimulation are indicated by blue (F_cold_) and red (F_hot_) bars; ΔF = ‖F_hot_–F_cold_‖. (**Right panel**) Individual ΔF values were plotted against their corresponding L-histidine concentrations. The fit of the data to Hill’s equation (red line) and the calculated Ec_50_ ± SD are shown.

A new functional approach based on microscale thermophoresis (MST) technology has recently been established in our laboratory to study the ligand-binding properties of SLC15 family members. In the initial work focusing on PepT1 (*SLC15A1*), a bacterial PepT1 orthologue from *Physcomitrella patens* was overexpressed in yeast, membrane preparations were isolated, and MST was used to assess the binding of PepT1 to some of its known substrates, such as the dipeptide glycylsarcosine (Gly-Sar) and the antiviral prodrug valacyclovir [34]. Following this work, MST was used to study the functional properties of human PHT1 (*SLC15A4*). To this end, a HEK293 cell line stably overexpressing PHT1 tagged with C-terminal GFP (hereafter referred to as PHT1-GFP OE) was generated. Cellular location and expression levels of PHT1-GFP were assessed by fluorescence imaging (Figure 1A). Imaging shows that the expression of PHT1-GFP is restricted to intracellular compartments. Notably, PHT1-GFP is predominantly observed surrounding Lysotracker (i.e., fluorescent dye staining acidic compartments such as lysosomes) stained vesicles. These results confirm the expression of PHT1-GFP in lysosomal membranes. Subsequently, membrane preparations of the PHT1-GFP OE cell line, comprising a mixture of cell membranes and intracellular vesicles, were isolated and used for MST measurements. The MST of PHT1-GFP was monitored by applying a temperature gradient in the presence of different substrate concentrations and/or buffer conditions. Representative MST traces recorded for the binding of a serial dilution of L-histidine to HEK293 PHT1-GFP OE membrane preparations are shown (Figure 1B). MST traces were used to calculate the dissociation constant (K_D_) for L-histidine, which was 4.1 ± 0.5 mM. In addition, binding properties were determined for a broad range of other potential PHT1 ligands, which will be published elsewhere, upon completion. These MST studies were the starting point of the present work; therefore, the same expression system has been used.

The main objective of the present work is to develop a transport assay that can be used to study the functional properties of PHT1, and moreover, serve as a tool for its pharmacological development. Secondly, it is expected that this assay will be instrumental in assessing whether the ligand-binding properties observed for PHT1 by MST or other binding methodologies represent a reflection of its actual transport activities. And therefore, allowing follow-up findings to emerge from such methodologies, including the validation of new substrates and potential modulators of PHT1. Since PHT1 has been reasonable well established as a H^+^-coupled symporter [7,8,10,11], its transport of substrates is expected to be electrogenic, i.e., a net translocation of positive charge should be associated with each transport cycle. Therefore, it was expected that SSME would provide an excellent method to study the transport function of PHT1, analogous to what has been successfully done for PepT1 [27]. 

### 3.1. SSME—A New Approach to Study the Functional Activity of PHT1 (SLC15A4)

**Figure 2:**
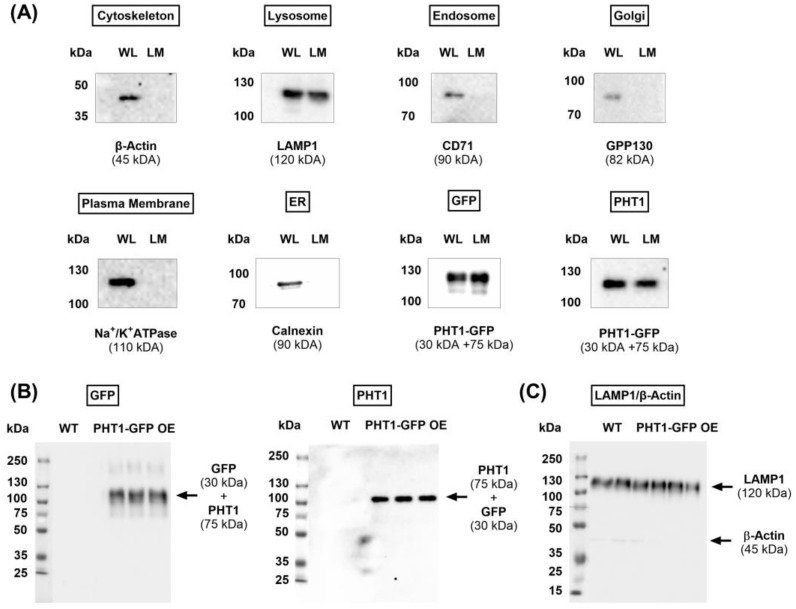
Western blot analysis of the expression levels of subcellular markers and PH1-GFP. (**A**) Representative blots showing the protein expression of the indicated subcellular markers in whole-cell lysate (WL) and lysosomal membrane preparations isolated from the PHT1-GFP OE HEK293 cell line. (**B**) Representative blots showing protein expression of GFP (**left panel**), PHT1 (**middle panel**) and LAMP1 and β-actin (**C**) in lysosomal membrane preparations isolated from the indicated cell lines. Expected protein size for each target is indicated between brackets. Protein ladder was overlaid in whole-blot images (**B**) to illustrate the size of the detected bands. Original Western blot images are available in Supplementary Materials.

SSME uses a coated gold sensor to physically absorb membrane fractions or vesicles that should contain large amounts of the protein under study. To obtain such membrane preparations, since PHT1 is found in intracellular compartments, a protocol to isolate lysosomal membranes was followed. Briefly, large amounts of HEK293 cells, wild type (hereinafter referred to as WT) or PHT1-GFP OE, were disaggregated by sonication and the corresponding lysosomal fractions were isolated by a series of ultracentrifugation steps, as described elsewhere [35]. As a control for the lysosomal membrane isolation protocol, the expression of GFP and PHT1, along with several cellular markers including β-actin (cytoskeleton), Lamp-1 (lysosome), CD71 (endosome), GPP130 (Golgi), Na^+^/K^+^-ATPase (plasma membrane), and Calnexin (ER), were compared between whole-cell lysate (WL) and lysosomal membrane preparations (LM) obtained from HEK293 PHT1-GFP OE cells (Figure 2A). As expected, in contrast to the whole-cell lysate, lysosomal membrane preparations were exclusively positive for GFP, PHT1, and LAMP1 expression, thereby confirming the successful isolation of lysosomal membranes overexpressing PHT1-GFP. PHT1-GFP expression in the WT and PHT1-GFP OE lysosomal membrane preparations was assessed by Western blot analysis using anti-GFP and anti-PHT1 antibodies (Figure 2B). Furthermore, expression levels of the cytosolic marker β-actin and the lysosomal marker LAMP1 were also determined as internal negative and positive controls for purity assessment of the lysosomal membrane isolation procedure (Figure 2C). The lack of immunodetection of GFP and PHT1 in the WT lysosomal membrane preparations indicates that there is no cross-contamination with PHT1-GFP or endogenous expression of PHT1. Therefore, the isolated WT lysosomal membranes provided a suitable negative control to study the functional activity of PHT1 by SSME.

**Figure 3:**
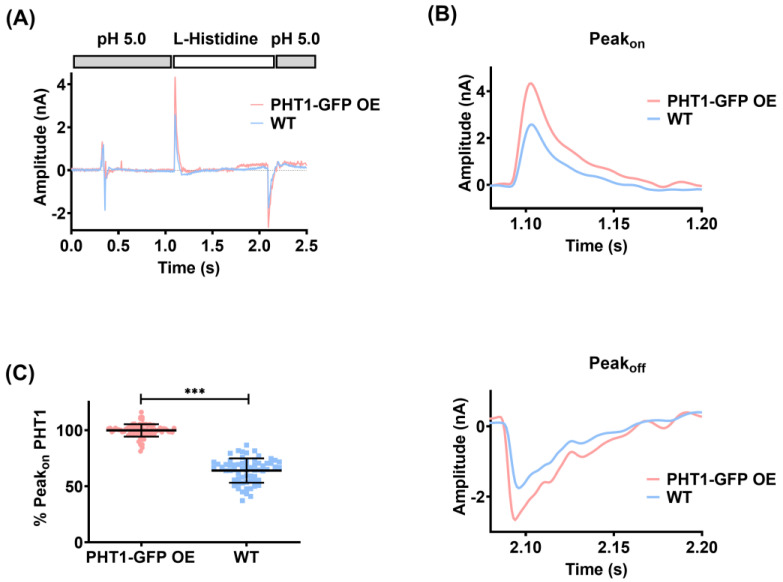
Assement of PHT1-GFP functional activity by solid-supported membrane-based electrophysiology (SSME). (**A**) Current traces recorded with gold sensors loaded with lysosomal fractions isolated from the indicated cell lines using a single solution exchange protocol BAB. Perfusion of non-activating (**B**) and activating solution (**A**) is indicated at the top of the graph by grey (**B**) and white (**A**) squares, respectively. The non-activating solution (**B**) is 10 mM citric acid pH 5.0 buffer, while activating solution (**A**) is solution B supplemented with 10 mM L-histidine. (**B**) Close-up view of Peak_on_ and Peak_off_ currents shown in C. (**C**) Statistical comparison of the mean ± SD of normalized Peak_on_ currents recorded with gold sensors loaded with lysosomal fractions isolated from HEK293 PHT1-GFP OE (red dots; N = 83) or HEK293 WT cells (blue squares; N = 64). ***; corresponds to *p* < 0.001.

To perform SSME recordings, PHT1-GFP OE lysosomal membranes were placed on coated gold sensors and mounted into the SURFER^2^R N1 SSME device. Activation of the PHT1-GFP transport activity is achieved by rapid solution exchange of its substrate L-histidine in a buffer that provides the H^+^ required for H^+^-coupled histidine transport (Figure 3A). The H^+^ influx into the lysosomal membrane vesicles charges the gold sensor by capacitive coupling, which generates an on-peak (Peak_on_). Washout of L-histidine by perfusion of L-histidine-free buffer through the gold sensor suppresses the H^+^ influx and generates the off-peak (Peak_off_). The observed asymmetry between the Peak_on_ and Peak_off_ could reflect different transport kinetics for influx and efflux transport processes (Figure 3B). Alternatively, it could be a consequence of differences in the driving force for each reaction. Peak_on_ is induced by the concentration gradient in the absence of a membrane potential, whereas Peak_off_ is induced by a different concentration gradient and the membrane potential that was generated through H^+^/L-histidine influx. The measurements were performed at pH 5.0, so the net charge of L-histidine was +1. Thus, the charge transfer to the sensors due to PHT1-mediated transport is the result of the contribution of both H^+^ and L-histidine. Likewise, non-specific currents might arise from the direct interaction of L-histidine with the membrane surface or from transport via other endogenous transporters present in the lysosomal membranes. 

To determine the contribution of non-specific currents to the electrical signal induced by PHT1 transport, the same recordings were performed with the lysosomal membranes isolated from WT cells. On-peaks recorded with WT lysosomal membranes upon L-histidine perfusion were on average ~40% lower than those observed for PHT1-GFP OE lysosomal membranes (Figure 3C). Overall, these results show for the first time electrophysiological recordings for PHT1-mediated transport, and although the PHT1 signal-to-background ratio was not optimal, there is a statistically significant and consistent difference between PHT1-induced and background signals supporting the suitability of the described SSME method to study the functional activity of PHT1.

### 3.2. Functional Characterization of PHT1 (SLC15A4) by SSME

**Figure 4:**
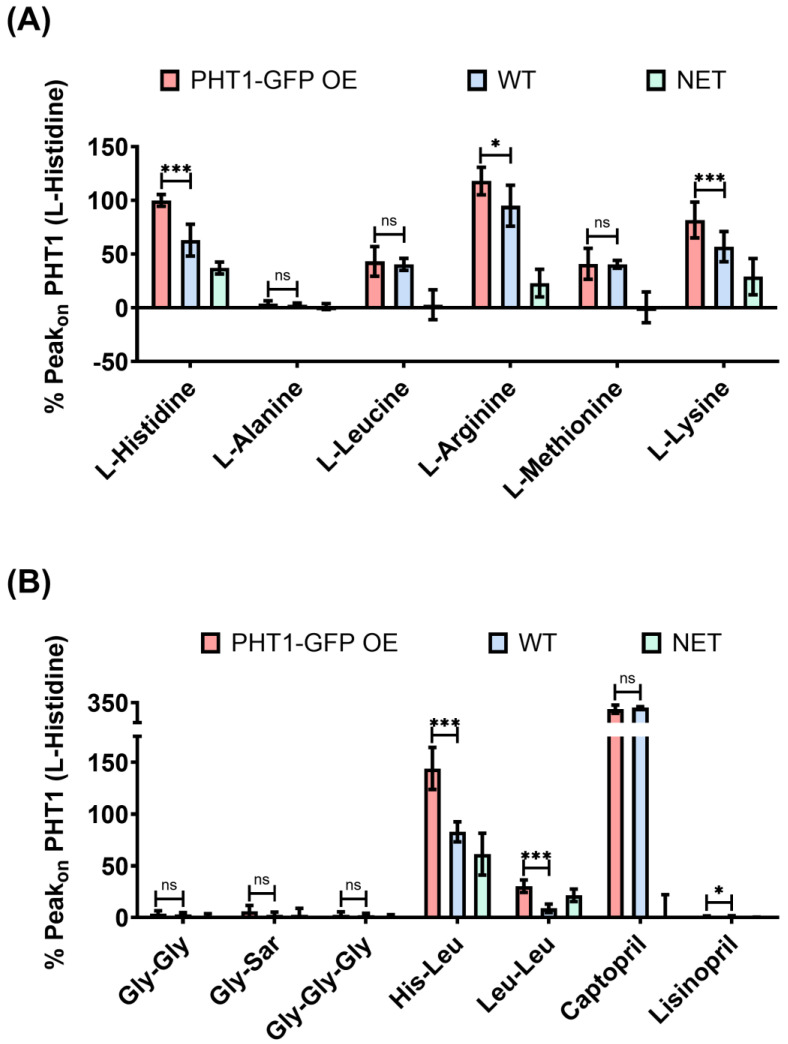
Substrate selectivity of PHT1-GFP by SSME. Statistical comparison of the mean ± SD Peak_on_ currents recorded for a series of AAs (**A**) and peptides (**B**) perfused at a concentration of 10 mM. To average currents recorded on different gold sensors Peak_on_ currents were normalized to the L-histidine current. Recordings were obtained with gold sensors loaded with lysosomal fractions isolated from HEK293 PHT1-GFP OE (red bars) or HEK293 WT cells (blue bars). The Net current for each substrate (green bars) was calculated by subtracting the mean Peak_on_ current calculated for the WT cells to each of the Peak_on_ currents recorded for the same substrate with the HEK293 PHT1-GFP OE cells. Statistical significance levels are indicated as follows: ns; *p* > 0.05; *; *p* < 0.05; ***; *p* < 0.001.

To assess the substrate selectivity of PHT1, sensors loaded with PHT1-GFP OE or WT lysosomal membrane preparations were perfused with a series of AAs (L-histidine, L-alanine, L-leucine, L-arginine, L-methionine, and L-lysine), di- and tripeptides (Gly-Gly, Gly-Sar, Gly-Gly-Gly, His-leu, and Leu-Leu), and peptidomimetic drugs (Captopril and Lisinopril), some of which have been previously proposed as PHT1 substrates [3,7,8], and Peak_on_ currents were recorded with the SURFER^2^R N1 device. Interestingly, when comparing the PHT1-GFP OE and WT Peak_on_ currents induced by AAs (Figure 4A), only the positively charged AAs (L-histidine, L-arginine, L-lysine) induced significant differences, while the uncharged AAs did not induce any current (L-alanine) or were the same for both lysosomal membrane preparations (L-leucine and L-methionine). The presence of large non-specific currents (WT Peak_on_) for some of these AAs suggests that there was an endogenous AAs transport system in the isolated lysosomal membrane preparations contributing to the recorded currents. 

Regarding the Peak_on_ currents induced by perfusion of di- and tripeptides (Figure 4B), glycine-containing peptides (Gly-Gly, Gly-Sar and Gly-Gly-Gly) did not elicit a significant response, whereas leucine-containing peptides (His-Leu and Leu-Leu) were electrogenic and induced currents with significant differences between both PHT1-GFP OE and WT experimental groups. Interestingly, the di-peptide Leu-Leu, despite being an uncharged substrate, was able to induce specific currents, a consequence of H^+^-coupled transport. Given that the Leu-Leu-induced currents were exclusively due to H^+^ influx, this could explain why the Leu-Leu-induced currents were lower than those observed for the charged substrates (L-histidine, L-arginine, L-lysine, and His-Leu), and furthermore provided the first direct evidence for H^+^ cotransport by PHT1. In contrast to the results obtained with the AAs, the di- and tripeptide-induced currents had much lower background signal levels. This probably reflects the lack of an endogenous transport system for these substrates in the isolated lysosomal membrane preparations. Surprisingly, peptidomimetic drugs (Captopril and Lisinopril) did not induce a clear specific activity (Figure 2B). Lisinopril induced very small currents that were statistically different between PHT1-GFP OE and WT; however, due to the reduced PHT1 signal-to-background ratio, it was not possible to investigate further its transport properties. Conversely, Captopril induced very large and unspecific currents. 

**Figure 5:**
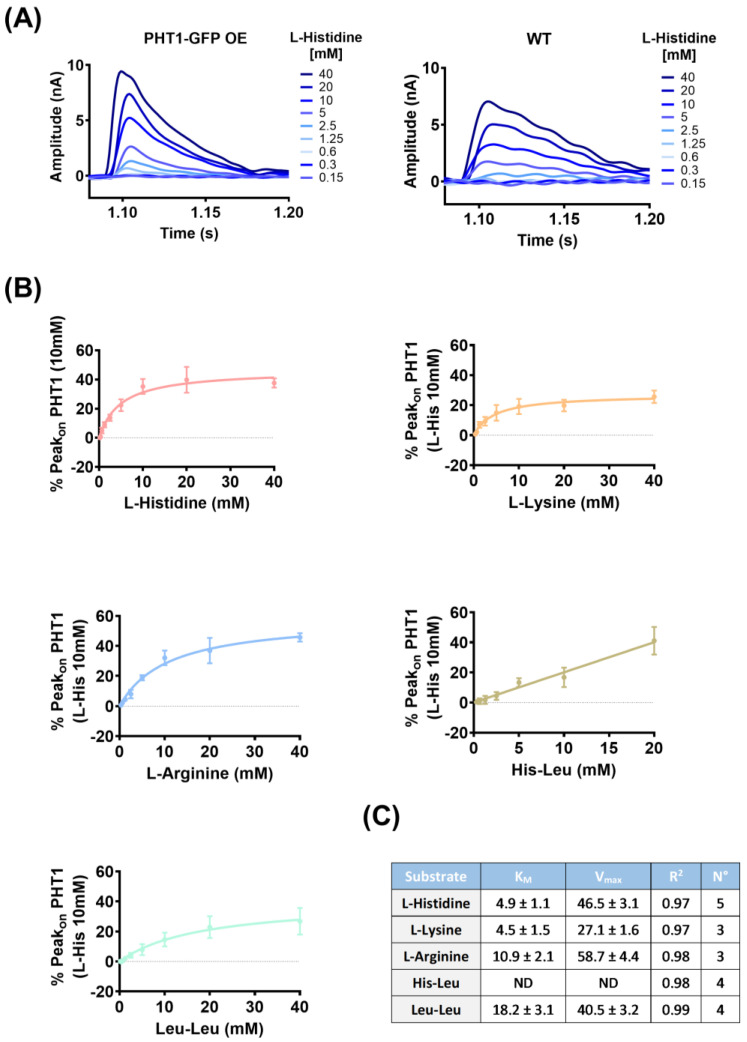
Identified PHT1-GFP substrate transport kinetics by SSME. (**A**) Representative Peak_on_ currents recorded at the indicated L-histidine concentrations [mM] with sensors loaded with lysosomal fractions isolated from the indicated cell lines. (**B**) Graphs showing the fit (solid line) of the Net Peak_on_ currents calculated for each of the indicated substrates to the Michalis–Menten equation. (**C**) Table showing the parameters calculated with the Michalis–Menten equation for the indicated substrates.

For kinetic studies, AAs and dipeptides that showed significantly different Peak_on_ currents between PHT1-GFP OE and WT lysosomal membrane preparations (Figure 4A,B) were selected. Peak_on_ currents were induced by concentration jumps of a serial dilution of the indicated substrates (0.15–40 mM) and recorded on the same gold sensors loaded with PHT1-GFP OE or WT lysosomal membrane preparations (Figure 5A). To eliminate the contribution of non-specific background currents (Peak_on_ WT) to the PHT1-GFP OE Peak_on_ recordings, Net Peak_on_ currents were calculated for each of the substrates (L-histidine, L-arginine, L-lysine, His-Leu, and Leu-leu) and fitted to the Michalis–Menten equation (Figure 5B), and the obtained kinetic parameters are shown (Figure 5C). It is noteworthy that the calculated K_M_ value for L-histidine, 4.9 ± 1.1 mM, is close to the E_C50_ value determined by MST (Figure 1A), which is 3.8 ± 0.1 mM. Regarding the other substrates, K_M_ values for L-lysine and L-arginine were also in the low millimolar range, whereas the K_M_ for Leu-Leu was 3–4 times higher. The K_M_ for His-Leu could not be determined because the calculated Net Peak_on_ currents did not saturate in the range of concentrations tested. Higher concentrations of His-Leu could not be recorded because the magnitude of the currents was outside the sensitivity range of the gain settings used in these experiments.

**Figure 6:**
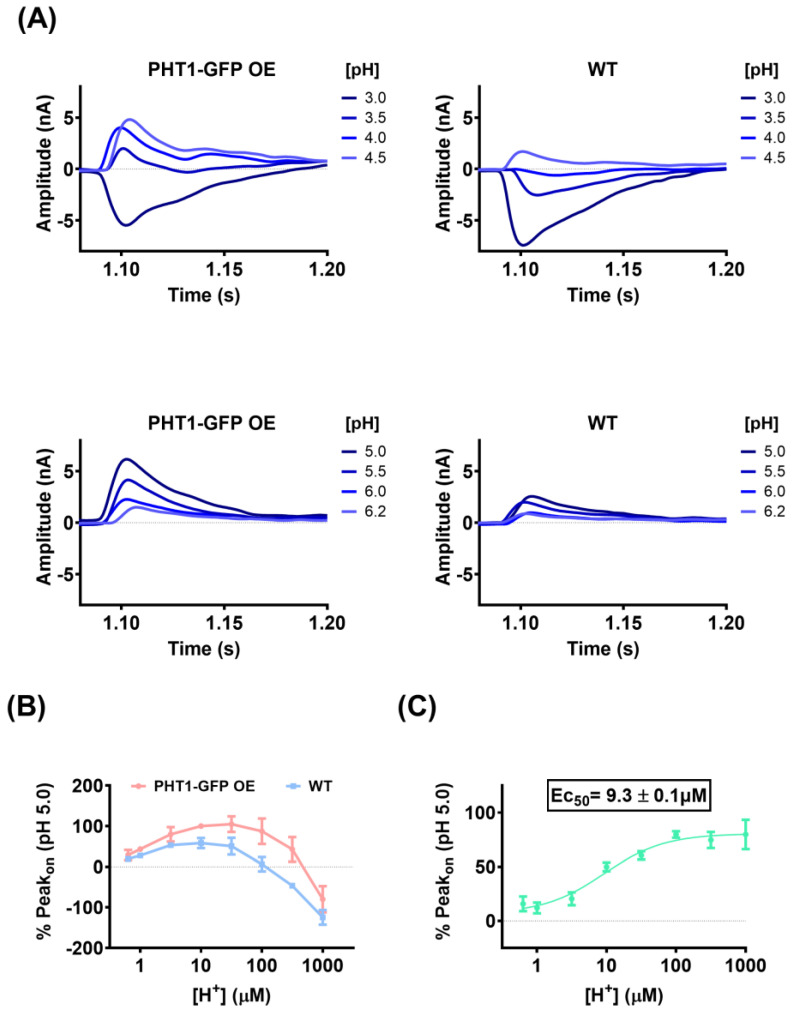
pH dependence of PHT1-GFP by SSME. (**A**) Representative Peak_on_ currents recorded for 10 mM L-histidine at the indicated extracellular pH. Recordings were obtained with gold sensors loaded with lysosomal fractions isolated from the indicated cell lines. (**B**) Mean ± SD Peak_on_ currents recorded for 10 mM L-histidine at the indicated extracellular H^+^ concentrations [µM]. To average currents recorded on different gold sensors, Peak_on_ currents were normalized to the current recorded for L-histidine 10 mM at extracellular pH 5.0. (**C**) Graphs showing the fit (solid line) of the Net currents calculated for each of the indicated extracellular H^+^ concentrations [µM] to a 4-parameter sigmoidal equation; calculated EC_50_ ± SD is shown in the graph. The Net Peak_on_ current for each extracellular H^+^ concentration was calculated by subtracting the Mean Peak_on_ current calculated for the WT cells to each of the Peak_on_ currents recorded for the same extracellular H^+^ concentrations with the HEK293 PHT1-GFP OE cells.

The electrophysiological properties observed during SSME experiments are consistent with the H^+^-coupled symport mechanism previously proposed for PHT1 [7,8,10,11]. To better understand the impact of H^+^ electrochemical gradients on the functional activity of PHT1, sensors loaded with PHT1-GFP OE or WT lysosomal membrane preparations were used to record Peak_on_ currents upon perfusion of L-histidine in the presence of different extracellular H^+^ concentrations (Figure 6A). The direction and magnitude of the currents were strongly pH dependent for both experimental groups. Maximum current magnitudes for the PHT1-GFP OE sensors were reached in the pH range of 4.5 to 5.0, while for the WT sensors they were reached in the pH range of 4.5 to 5.5. Overall, the maximum current magnitudes were 40 to 50% higher with the PHT1-GFP OE sensors, in agreement with the previous experiments. Interestingly, at more acidic and alkaline extracellular pH conditions, the current magnitudes decreased in both experimental groups, showing a bell-shaped pH-dependence profile (Figure 6B). Surprisingly, under highly acidic extracellular conditions, the direction of the Peak_on_ current was reversed. The reverse Peak_on_ currents were larger and were observed for a wider range of acidic pH conditions for WT sensors (i.e., 3.0 to 3.5). These reverse currents are likely a consequence of non-specific currents mediated by transport systems present in the lysosomal membrane preparations that mask PHT1-GFP related currents. To avoid the bias introduced by the non-specific currents (Peak_on_ WT), net Peak_on_ currents were calculated and used to determine the concentration of H^+^ required to achieve half maximal activity (Ec_50_) of PHT1-GFP (Figure 6C). The calculated Ec_50_, 9.3 ± 0.13 µM, corresponds to a pH 5.03.

### 3.3. Molecular Docking—PHT1 (SLC15A4) cryo-EM and AlphaFold 3D Structures

**Figure 7:**
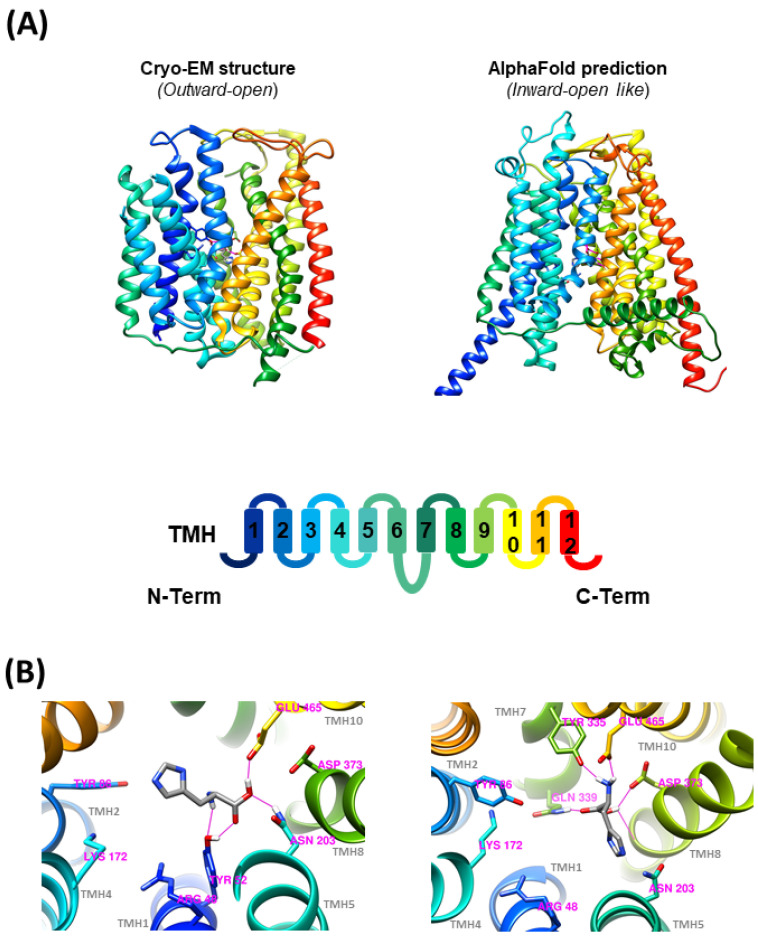
Molecular docking. (**A**) (**Upper panel**) Side view of the indicated 3D structures of human PHT1 (*SLC15A4*). (**Lower panel**) Schematic representation of the colour coding of the different transmembrane helices (TMH). (**B**) Detailed bottom-up view of L-histidine docking to (**left panel**) cryo-EM structure in an outward-open state and (**right panel**) AlphaFold structure prediction in an inward-open like state. Side chain of the amino acids predicted to take part in substrate binding and the amino acids forming H-bonds (pink lines) with L-histidine are shown.

To confront the substrate selectivity results (Figure 4) with an orthogonal method, the potential PHT1 substrates studied by SSME were docked into the human PHT1 3D structure obtained by cryo-EM (Figure 7A; left) or predicted by AlphaFold (Figure 7A; right). Structure energy minimization after molecular docking revealed the formation of several H-bonds between L-histidine and various AA side chains present in the docking box defined within each of the PHT1 structures. In the case of the outward-open cryo-EM structure, L-histidine formed H-bonds with residues Y52, N203, and E465 (Figure 7B; left). In contrast, L-histidine docking with the inward open-like structure predicted by Alpha-Fold revealed the formation of H-bonds with residues Y335, N339, E465, and D373 (Figure 7B; right). Members of the SLC15 family have several highly conserved residues proposed to be involved in peptide binding. In the case of human PHT1, R48, Y86, K172, N203, D373, and E465 are potentially required for interactions with both C- and N-termini of their substrate peptides [2]. Meanwhile, residues W332, Y335, F492, and S496 could be relevant for interactions with the substrate peptide side chains [2]. Interestingly, the molecular docking experiments showed direct interaction of L-histidine with several of these residues, supporting the reliability of the best-scoring docking conformations presented here (Figure 7B). Subsequently, molecular docking experiments were conducted under the same conditions with the other potential PHT1 substrates previously evaluated by SSME. The best docking scores, the number of H-Bonds and the residues involved in H-Bond formation are summarized in Table 1. Among the single AAs, L-histidine, L-arginine, and L-lysine showed the best docking scores. For di- and tripeptides, the best scores were obtained for His-Leu and Leu-Leu. For peptidomimetic drugs, Lisinopril had the best score. Overall, the best scoring substrates within each substrate type, Lisinopril, His-Leu, Leu-Leu, L-arginine, L-histidine, and L-lysine were the substrates identified in the SSME experiments.

To assess the influence of pH on the molecular docking of PHT1 and its proposed substrates, the protonation state of human PHT1 3D structures was adjusted to pH 5.0, which is close to the physiological conditions under which PHT1 operates. Molecular docking experiments were then repeated. The main characteristics of the best scoring ligand docking conformations identified are presented in Table 2. A comparison of the results of the molecular docking experiments carried out using the standard protonation protocol (adjusted to pH 7.0) (Table 1) with those carried out adjusting the pH to 5.0 showed that the highest docking scores for amino acids, di- and tripeptides and drugs exhibited identical trends in both protonation states. However, at pH 5.0 the docking scores were slightly higher, especially for the SSME-identified substrates. In contrast, some discrepancies were observed in the number of H-Bonds and the residues involved in their formation. Nevertheless, the observed results indicate that the PHT1 binding preferences determined by molecular docking are consistent at both protonation states. The higher binding scores observed at pH 5.0 are likely to reflect the involvement of H^+^ as a co-substrate facilitating PHT1 transport activity.

### 3.4. Molecular Dynamic (MD) Simulations—PHT1 (SLC15A4) Cryo-EM and AlphaFold 3D Structures

**Figure 8:**
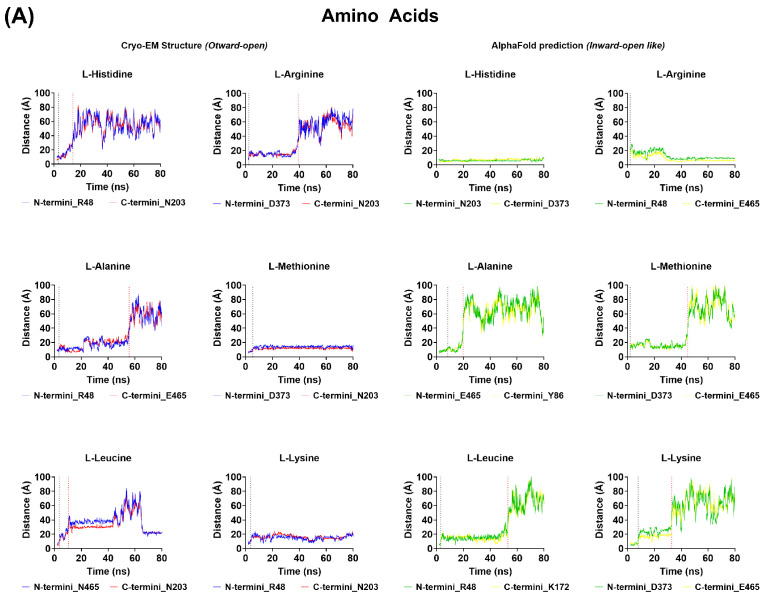

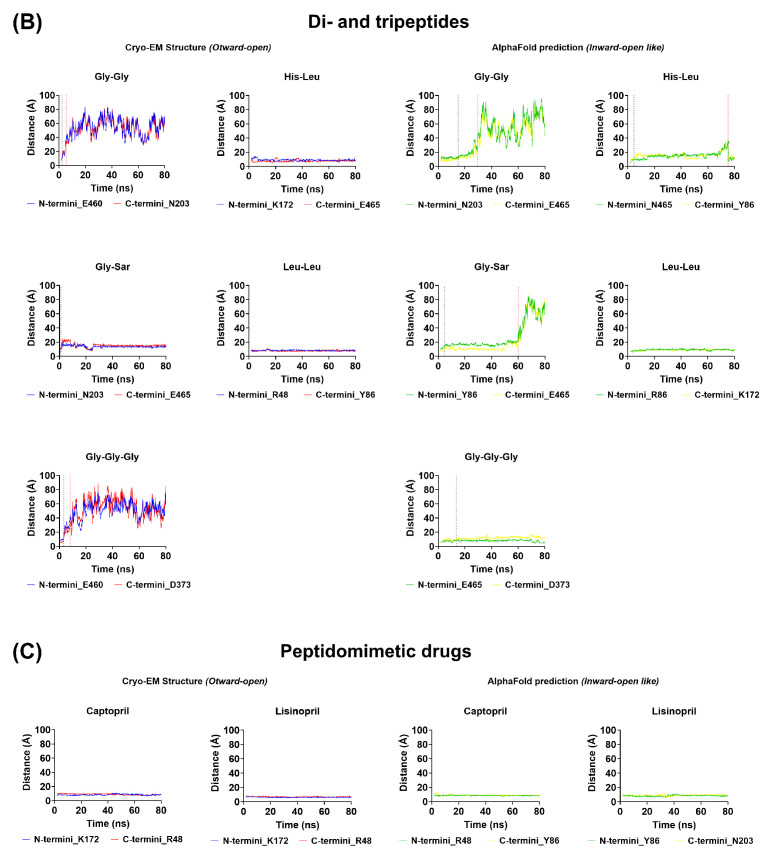
Molecular Dynamics (MD) simulations. The stability of the initial docking conformation was assessed by monitoring the changes in distance between the C- and N-termini of the docked AA (**A**), di-tri-peptide (**B**) and peptidomimetic drugs (**C**) and the indicated AA residues present in the PHT1 binding site. The dotted lines indicate the time point at which the molecules exited their initial docking binding site (black dotted line) or the entire protein structure (red dotted lines). The distances measured over the simulation time with molecules docked to the PHT1 cryo-EM structure are colored red (N-termini) or blue (C-termini), while for the molecules docked to the AlphaFold predicted structure are colored green (N-termini) or yellow (C-termini).

To validate the observations made by molecular docking, the stability of the best-scoring conformations determined for each of the potential PHT1 substrates was assessed by MD simulations. Briefly, substrate-docked cryo-EM and AlphaFold human PHT1 3D structures were embedded in POPC membranes in the presence of 150 mM NaCl, and MD simulations were conducted for 150 ns. Observed docking stability (ns) (i.e., time spent in the initial docking conformation) and total interaction time (ns) (i.e., time spent in contact with PHT1) were quantified and the results are presented in Table 3. To calculate these parameters, spheres encompassing the corresponding binding sites (5Å radius) and the entire protein (30 Å radius) were defined and the amount of time (ns) that at least 75% of each potential PHT1 substrate spent in these locations throughout the simulation was determined. In addition, to illustrate the changes in ligand position observed during the MD simulations, the distance (Å) between the C- and N-termini of the potential PHT1 substrates and the indicated AA residue part of the PHT1 binding site (Figure 7B) throughout the simulation are shown (Figure 8). Simulation systems remained stable throughout the 150 ns and no alpha–helix denaturation nor lipid penetration into the proteins were observed. The MD simulations conducted for each potential PHT1 substrate were found to be consistent between the cryo-EM and AlphaFold human PHT1 substrate-docked structures for L-alanine, L-leucine, Gly-Gly, Leu-Leu, Captopril, and Lisinopril. However, different patterns were observed for the rest of the molecules. Among the docked single AAs, L-histidine, L-methionine, L-lysine, and L-arginine remained near their initial docking conformations for the majority of the duration of at least one of the two simulations. However, in the rest of the simulations conducted with single AAs, after 2 to 8 ns, the molecules left their initial docking conformations and began to move within the protein. In most cases, the molecules exited the protein after 10 to 50 ns. In the case of di- and tripeptides, Leu-Leu remained in the initial docking conformation throughout the course of both simulations. Similarly, His-Leu remained in the initial docking conformation throughout at least one of the two simulations. In contrast, Gly-Gly remained only 2 to 14 ns in its initial docking conformation and after 5 to 30 ns exited the protein. Similarly, Gly-Sar and Gly-Gly-Gly did not remain for an extended period in the initial docking conformation, 1 to 13 ns, but remained within the protein structure throughout at least one of the two simulations. In the other simulations, the molecules left the protein after 8 to 60 ns. For the peptidomimetic drugs, both Captopril and Lisinopril remained in the initial docking conformation for the entire duration of their respective simulations.

Overall, the MD simulations provide support for the molecular docking and SSME experiments in that L-histidine, L-arginine, L-lysine, His-Leu, Leu-Leu, and Lisinopril are PHT1 substrates, since at least in one of the two simulations, these molecules remained in close proximity to the initial docking conformation for the majority of the duration of the simulation. Moreover, in accordance with the SSME experiments, L-alanine, L-leucine, and Gly-Gly do not appear to be substrates of PHT1, as evidenced by the fact that in all simulations the molecules left the protein in the initial 5 to 55 ns. This is also consistent with the low molecular docking scores obtained for these substrates. The outcomes of the simulations with L-methionine, Gly-Sar, Gly-Gly-Gly, and Captopril are open to various interpretations. 

## 4. Discussion

Previous studies have attempted to characterize the functional properties of PHT1 (*SLC15A4*) but the results are inconsistent in terms of substrate selectivity, transport kinetics of the identified substrates, and pH-dependence [2,3,7,8,10,11]. One of the reasons for this variability is the intracellular location of PHT1, which has hindered the interpretation of the results obtained with standard cell-based transport assays, designed for transporters expressed in the plasma membranes [10,11]. To overcome this problem, several strategies have been employed, including the generation of genetic variants of PHT1 with the goal of targeting protein expression to the plasma membrane [8], the use of *Xenopus laevis* oocytes as expression system [7], or the direct use of the purified protein [2]. However, the functional characterization of PHT1 was still inconsistent among the different studies, making it difficult to decide which one is the most reliable. In the present work, we introduce a new transport assay, which allowed us to study the functional activity of PHT1 directly from isolated lysosomal membranes (Figure 3). This is a major advantage because it avoids harsh protein purification protocols that could compromise protein integrity, as well as the use of non-physiologically relevant overexpression systems. In addition, it provides the correct membrane lipid composition and possible protein expression partners, factors that likely influence the functional behavior of PHT1. 

Regarding the methods used to study the functional activity of PHT1, previous studies used radiolabeled substrate transport assays as a readout of PHT1 functional activity [3,7,8,10,11]. This classic transport assay is highly sensitive and very well-established; however, it requires specific radiolabeled substrates and the expression of the target protein on the plasma membrane. In contrast, a recent study assessed the binding of a of potential substrates to PHT1 by a thermal shift assay (TSA) using purified chicken PHT1. TSA experiments do not necessarily require expression of the target on the plasma membrane but do require validation by a direct transport assay of the observed target-ligand interactions. Moreover, while TSA experiments showed interactions with lysine- and arginine-containing peptides and even TASL, they failed to show with free AAs, including the major PHT1 substrate L-histidine, nor with histidine-containing peptides, nor with other PHT1 substrates such as carnosine or MDP [2]. In contrast to all these previous works, in the present study, the functional activity of PHT1 is measured in its native lysosomal environment by SSME. This method has been successfully applied to study the functional properties and pharmacology of other lysosomal channels and transporters such as the cationic channel TMEM175 [36] or the Cl^-^/H^+^ exchanger CLC-7 [37]. In the present study, the SSME method allowed for the first time to measure the electrogenic properties of PHT1, which are related to the H^+^-driven cotransport mechanism characteristic of SLC15 family members [1]. In addition, SSME was used to determine the substrate selectivity among a series of AAs, di- and tripeptides, and peptidomimetic drugs, the kinetic properties of the identified substrates and the pH-dependence were determined. As expected, our results present certain discrepancies with previous studies, which are presented and discussed below.

### 4.1. Substrate Selectivity 

SSME recordings revealed a binding affinity for L-histidine in the low millimolar range, K_M_ = 4.9 ± 1.1 mM (Figure 5), which was close to the affinity determined by MST, K_D_ = 4.1 ± 0.5 mM (Figure 1B). In contrast, previous work using the *Xenopus laevis* oocytes microinjected with WT rat PHT1 [7] or MDCK cells overexpressing a mutant variant of human PHT1, which shifted expression from the subcellular to the cell membrane [8], showed affinities for L-histidine in the low micromolar range, K_M_ ≈ 16–17 µM. The reason for this difference is unclear, however, in the latter case, both studies implied unnatural overexpression at the plasma membrane, where this transporter may behave differently compared to the endosomal/lysosomal environment. Surprisingly, studies using *Xenopus laevis* oocytes did not assess the membrane expression of PHT1 or show the endogenous levels of L-histidine transport in non-injected oocytes. Similarly, studies using MDCK cells overexpressing a mutant variant of PHT1 did not account for the contribution of the non-specific signal to their K_M_ calculations. In any case, consistent with the results presented here, other lysosomal AA transporters, such as cystinosin (*SLC66A4*), PAT1 *(SLC36A1*) and SNAT9 (*SLC38A9*) have affinities for their respective substrate AAs, L-cysteine (K_M_ = 1.5 mM) [38], L-proline (K_M_ = 1.8 mM) [39] and L-arginine (K_M_ = 2.7 mM) [40] in the low millimolar range, supporting the results presented here using both SSME and MST. 

Substrate selectivity experiments using SSME led to the identification of several novel PHT1 substrates such as L-arginine, L-lysine, His-Leu, and Leu-Leu (Figure 4), and defined for the first time their transport kinetics (Figure 5). Among these substrates, the His-Leu and Leu-Leu dipeptides have previously been proposed as PHT1 substrates by competition experiments with radiolabeled L-histidine [7] and direct binding by thermal shift assay [2], respectively. L-arginine and L-lysine have been identified for the first time as PHT1 substrates in the present study. Moreover, consistent with our findings, competition experiments with radiolabeled L-histidine also showed that L-leucine and L-methionine are not PHT1 substrates [7]. In contrast, substrates such as Gly-Sar, Gly-Gly, Gly-Gly-Gly, and Lisinopril, previously reported to be PHT1 substrates [7,8], did not show relevant electrogenic activity in the SSME recordings. 

Notably, for some of the tested substrates, especially the single AAs and Captopril, large unspecific currents were recorded. In this regard, several solute carriers (SLCs) have been reported to be involved in the transport of AAs across lysosomal membranes, some of which could potentially contribute to these non-specific currents. Possible candidates include, SLC38A9 [41,42], LYAAT1 (*SLC36A1*) [41,43], PQLC2/LAAT1 (*SLC66A*1) [41,44], and SNAT7 (*SLC38A7*) [41,45], and less likely, as it is mainly expressed in immune cells, PHT2 (*SLC15A3*) [46]. Regarding Captopril-induced non-specific currents, it has been shown that certain drug efflux transporters, such as the ABC transporter TAPL (*ABCB9*), may be involved in peptide transport across lysosomal membranes [47,48]. However, it is unknown whether TAPL-mediated peptide transport is electrogenic and whether Captopril is a substrate of TAPL or whether other lysosomal drug efflux transporters are involved. Thus, the origin of such non-specific currents is not clear. 

### 4.2. pH-Dependence 

In agreement with previous studies [7,8,10], PHT1-mediated transport was highly dependent on the driving force provided by H^+^ electrochemical gradients (Figure 6). Furthermore, pH-dependency studies by SSME revealed that half-maximal PHT1 activity is reached at pH~5.03, which is close to the optimal lysosomal pH for hydrolase activity (pH ≈ 4.6) [49]. It is worth mentioning that the pK_a_ of L-histidine is ~6, thus, the difference in one pH unit between L-histidine pK_a_ and the calculated E_C50_ for PHT1 supports the specificity of the recorded currents. Conversely, in the case of the WT sensors, the observed pH dependence could be only a reflection of the protonation state of L-histidine interacting with the membrane surface or the activity of other H^+^-transport systems present in the lysosomal membranes. 

The pH-dependence of PHT1 determined by SSME exhibited a bell-shaped profile, similar to the profiles observed for other H^+^-coupled symporters, such as the bacterial sugar transporters, lactose permease (*LacY*), fucose permease (*FucP*) and xylose permease (*XylE*) [50]. However, it was surprising that under highly acidic extracellular conditions, the current direction was reversed for both PHT1-GFP OE and WT sensors. This suggests that under these pH conditions, charged elements such as H^+^, L-histidine, reorientation of charged elements embedded in the membrane, or other unanticipated charged elements present in the buffer solution move across the lysosomal membranes resulting in opposite membrane polarization. The origin of these reverse currents remains unclear, the presence of PHT1-GFP in the lysosomal membrane reduced the magnitude of the reverse polarization effect. This was probably a consequence of PHT1-GFP-mediated transport of H^+^ and L-histidine into the lysosomal membranes, which generates a current in the opposite direction to that recorded. 

Many ionic channels and SLCs are expressed in lysosomal membranes [49], some of which could be the source origin of the non-specific currents recorded. Possible candidates to contribute to the non-specific currents induced by H^+^ electrochemical gradients induced currents include the lysosomal H^+^ pump [51], the voltage-gated H^+^ channel [52] or lysosomal Cl^−^/H^+^ exchangers such as CLC-7 [37]. 

Finally, it is worth mentioning that SSME recordings with the uncharged substrate Leu-Leu provided the first direct evidence of H^+^ transport through PHT1. 

### 4.3. Molecular Docking

To contrast the substrate selectivity results obtained by SSME (Figure 4), potential PHT1 substrates tested by SSME were docked into the cryo-EM and AlphaFold predicted human PHT1 structures. The cryo-EM structure was obtained in an outward-open conformation (Figure 7A; left), while the structure predicted by AlphaFold (Figure 7A; right) resembles an inward open-like conformation, as the binding site is accessible exclusively from the cytosolic side. Therefore, molecular docking provided insight into the differences between the atomic interactions of L-histidine and the amino acid sidechains involved in substrate binding in both outward-open (Figure 7B; left) and inward-open (Figure 7B; right) conformations. 

Overall, the molecular docking experiments with both PHT1 structures showed direct interaction of L-histidine with several of the amino acid residues described as key for transport in PHT1 [2], supporting the reliability of the best scoring docking conformations shown. However, the orientation, H-bond, and precise location of the L-histidine docking show differences between the two structural models. Interestingly, in both cases, there is a direct interaction of the L-histidine with E465, a residue that has been shown to be essential for the binding of TASL to PHT1 [17]. Moreover, in the inward-open-like conformation of the AlphaFold structure, the proximity of TMH7 to the binding site leads to the formation of additional H-Bonds, which may partially explain the observed conformational differences. In this regard, the reorientation of the substrate towards the exit pathway, observed in the inward-open-like docked conformation, could be a necessary step for substrate release. 

Interestingly, the scores obtained during the molecular docking of the other potential PHT1 substrates support the substrate selectivity determined by SSME. However, the docking scores did not correlate with the calculated KM or substrate preferences determined by SSME. This could be consequence of the size of the substrates, as a larger molecule can potentially establish more interactions, and thus receive higher scores, as most scoring functions are additive [53].

Given that PHT1 is an H^+^-coupled transporter, to assess potential pH-related bias in the determination of substrate binding preferences, PHT1 structures were adjusted to protonation states corresponding to pH 7.0 (i.e., standard procedure) and 5.0 prior to performing molecular docking experiments. As shown in Table 1 and Table 2, no significant differences in substrate binding preferences were observed between both protonation states. Conversely, the number of H-bonds and the residues involved were more variable. Nevertheless, H-bonds with important substrate-binding residues such as N203 and E465 in the outward-open structure and residues in TMH7 of the inward-open-like structure such as Y355 were frequently observed for both protonation states. It is also noteworthy that docking scores were in general higher for structures adjusted to pH 5.0, likely reflecting the involvement of H^+^ as a co-substrate facilitating additional substrate binding to PHT1. 

Molecular Dynamics (MD) simulations were conducted to evaluate the stability of the best-scoring docking conformations. As illustrated in Table 3 and Figure 8, L-histidine, L-arginine, L-lysine, His-Leu, Leu-Leu, and Lisinopril remained in close proximity to the initial docking conformation for the majority of the duration of at least one of their respective simulations. In contrast, L-alanine, L-leucine and Gly-Gly exited the protein structure during the first 50 ns of their respective simulations. These findings are in accordance with the SSME experiments (Figure 4) and molecular docking predictions on the PHT1 substrate selectivity. It is noteworthy that some of the PHT1 substrates identified by SSME and validated by molecular docking, such as L-histidine, L-arginine, L-lysine, and His-Leu, did not remain in the initial docking conformation throughout both simulations. In one of the two simulations, these PHT1 substrates exited the protein structure approximately 10 to 70 ns after moving out of the initial docking conformation. These transitions may reflect the differences between PHT1 3D structures and their respective orientations, actual substrate transport events or simply that the identified best-score docking confirmations for these substrates were not optimal. These interpretations are beyond the scope of the present study.

### 4.4. PHT1 SSME Assay Limitations

Although several important aspects of the functional properties of PHT1 were revealed by SSME, the significant contribution of various non-specific signal sources to the recorded PHT1-GFP-OE currents remained the main limitation during this study. To overcome this problem, Net Peak_on_ currents were calculated prior to kinetic analysis. However, for some substrates, such as Lisinopril, the net Peak_on_ currents calculated for PHT1-GFP-OE were not sufficient to perform more detailed analyses. It is noteworthy that background signal levels were variable depending on the type of substrate and its charge. In general, charged substrates, such as the positively charged AAs, induced larger non-specific currents, suggesting a direct interaction of their charges with the membrane surface as main source of background signal. This seemed to be reflected in the higher degree of symmetry between Peak_on_ and Peak_off_ currents observed for the charged substrates in the WT sensors. Interestingly, non-charged AAs, di- and tripeptides, and peptidomimetic drugs showed different levels of non-specific signal. Given that different substrates were often tested on the same sensor and always at the same concentration, this is a clear indication of the presence of endogenous transport systems present in the isolated lysosomal membranes that contribute to the non-specific signal according to their own substrate preferences. In this context, some examples of lysosomal AA transporters, drug efflux transporters and H^+^ channels that might contribute to such signals have already been discussed. 

Overall, it seems more appropriate to perform the described SSME experiments in the native lysosomal environment of PHT1. However, this has the disadvantage of introducing a background signal due to the activity of other SLCs and ion channels that mask the actual transport activity of PHT1. In this regard, future studies following the SSME approach described here would benefit from improving PHT1 signal-to-background ratio. This would require increasing the density of PHT1 in the lysosomal membranes on the gold sensors. This could probably be achieved by improving lysosomal membrane isolation protocols (e.g., using lysosome immune-isolation methods, such as LysoIP [54]) or PHT1 overexpression strategies (e.g., knocking down endogenous lysosomal transporters responsible for the background signal). Alternatively, purified PHT1 reconstituted into proteoliposomes could be used. However, protein purification procedures are generally complex and may compromise the structural integrity of membrane proteins. Furthermore, they may also remove important accessory elements of PHT1 such as lipids or interacting proteins. 

## 5. Conclusions

This work presents a new functional assay based on the SSME methodology, which allows the study of human PHT1 in its native lysosomal environment. Using this new approach, a characterization of several functional properties of PHT1 was performed. Major findings include the first recordings of electrophysiological properties and direct evidence for H^+^-cotransport by PHT1, a detailed pH-dependency analysis, an assessment of PHT1 substrate selectivity among a variety of AAs and peptides, as well as transport kinetics for L-histidine and the PHT1 substrates L-arginine, L-lysine, His-Leu and Leu-Leu identified herein. Moreover, some of these observations were validated by orthogonal methods such as MST, molecular docking and MD simulations. 

Transport and scaffolding functions of PHT1 can influence the lysosomal environment [4,26], enable NOD and TLR signaling [3,13,14], and modulate mTOR activity in human immune cells [4,15]. In addition, PHT1 plays a central role in SLE pathogenesis [18,19,20,21,22]. Thus, major efforts are currently underway to identify molecules capable of modulating PHT1 function as strategy for the treatment of diseases associated with altered innate immune responses [55,56]. Despite the obvious interest in PHT1 as a drug target, the pharmacological development of PHT1 has been limited by the lack of appropriate functional assays and scarce structural information. Therefore, it is expected that the SSME-based assay introduced in the present work could serve as a screening platform to identify novel PHT1 modulators or to evaluate the pharmacological activity of newly found modulators [55,56]. Furthermore, this SSME assay can also be used to further characterize the functional properties of PHT1 or to study the impact of medically relevant SNPs [21] on normal PHT1 function, all of which would significantly contribute to a better understanding of its physiological role and therapeutic value as drug target. 

## Figures and Tables

**Table 1:** Table summarizing the docking, including the best score, number of H-bonds, and residues taking part in H-bonding in the best-scoring conformation.

Docking Substrate	Cryo-EM Structure			AlphaFold Prediction		
	Score	H-Bonds	Residues	Score	H-Bonds	Residues
*L-Histidine	−4.8	3(*3)	Y52,N203, Y199 or *E465	−4.3	1 (*5)	*Y335, *Q339 D373, *E465
L-Alanine	−3.5	1	N203	−3.3	4	Y86,Y335, E465(2)
L-Leucine	−4.3	3	Y52,N203, E465	−3.7	2	Q339, D373
L-Arginine	−5.2	7	Y199(2) N203, Y335, T370(2), E465,	−4.5	4	N203(2), Y335, D373
L-Methionine	−3.9	3	Y52,N203, E465	−3.5	2	T370,E465
L-Lysine	−4.4	3	N203, E465(2)	−4.1	2	Q339,T370
Gly-Gly	−4.1	3	Y52,N203, E465	−3.9	2	Y335,E465
Gly-Sar	−4.2	3	Y335, T370, E465	−3.7	5	Y86, Y335(2), Q339, D373
Gly-Gly-Gly	−5.0	3	N203, D373, E465	−5.0	6	Y86, E465(3), D373, Y335
His-Leu	−6.0	4	Y52, Y199(2), N203,	−5.6	3	D373, E465(2)
Leu-Leu	−5.4	3	N203, Q399(2)	−5.0	3	Y33,Y52 E465
Captopril	−4.8	2	N203, Q399	−4.5	1	N203
Lisinopril	−6.9	4	Y52,Y86, K172,N203	−6.1	2	Y86,E465

* Additional H-bonds revealed by structure energy minimization of the L-histidine PHT1 docking.

**Table 2:** Table summarizing the docking results obtained with structures adjusted to the protonation state corresponding to a pH of 5.0, including the best score, number of H-bonds, and residues taking part in H-bonding in the best-scoring conformation.

Docking Substrate	Cryo-EM Structure			AlphaFold Prediction		
	Score	H-Bonds	Residues	Score	H-Bonds	Residues
L-Histidine	−5.2	2	N203, D373	−4.7	2	Y335, D378
L-Alanine	−3.5	4	Y199, N203(2), E465	−3.3	2	Y335, D378
L-Leucine	−4.1	3	N203, Y335, Q339	−4.0	2	Y49, D378
L-Arginine	−5.2	6	Y52, N203, T370, Q339, E465(2)	−4.8	3	Y209, D378, E471
Methionine	−3.8	2	N203, Y335	−3.7	2	D378, E471
L-Lysine	−4.2	5	Y52, N203, Y335, Q339, E465	−4.2	2	D378(2)
Gly-Gly	−4.1	2	N203, E465	−4.2	5	Y335, D378, E471(3)
Gly-Sar	−4.2	3	N203, Q339, E465	−4.0	3	N213, T375, D378
Gly-Gly-Gly	−5.0	3	Q339, E465(2)	−5.1	3	R45, K182, D378
His-Leu	−6.2	4	R48, Y52, Q339, E465	−5.8	4	Y49, Y83, Y335, D378
Leu-Leu	−5.5	3	Y52, Y199, E465	−5.0	2	Y49, D378
Captopril	−5.0	3	N203, T370, E465	−4.5	2	Y335, D378
Lisinopril	−6.9	2	Y52, E465	−6.9	3	Y335, D378, S475

**Table 3:** Table summarizing the Molecular Dynamics (MD) simulation results, including docking stability (ns) and total interaction time (ns).

Docked Substrate	Cryo-EM Structure		AlphaFold Prediction	
	Docking Stability (ns)	Total Interaction Time (ns)	Docking Stability (ns)	Total Interaction Time (ns)
L-Histidine	3.38	14.09	84.48	93.78
L-Alanine	3.48	55.69	8.38	19.79
L-Leucine	3.48	10.48	3.08	53.19
L-Arginine	2.48	39.28	1.89	150
L-Methionine	5.49	150	1.89	44.79
L-Lysine	3.69	150	7.88	32.88
Gly-Gly	2.29	5.38	14.88	29.48
Gly-Sar	1.31	150	4.58	60.08
Gly-Gly-Gly	3.01	8.01	13.38	150
His-Leu	150	150	4.49	75.19
Leu-Leu	150	150	150	150
Captopril	150	150	150	150
Lisinopril	150	150	150	150

## Data Availability

The data presented in this study are available on request from the corresponding author.

## Supplementary Materials

The following supporting information can be downloaded at: https://www.mdpi.com/article/10.3390/biom14070771/s1, Original Western blot images.
