# Supplementary material for: Functional Characterization of the Lysosomal Peptide/Histidine Transporter PHT1 (SLC15A4) by Solid Supported Membrane Electrophysiology (SSME)
Source: Biomolecules. 2024 Jun 28;14(7):771. doi: 10.3390/biom14070771 (PMC11275134; doi:10.3390/biom14070771)
Supplement: Supplementary file 1 [file biomolecules-14-00771-s001.zip › biomolecules-3073090-supplementary.pdf]

GPP130

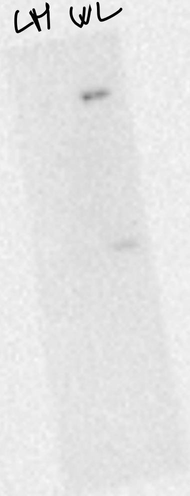

Fig. 2A

Fig. 2A

LAMP-1

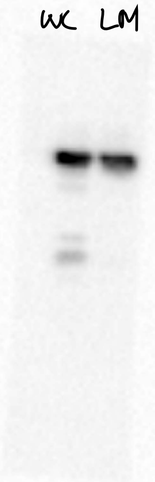

B- ACTIN Fig. 2A

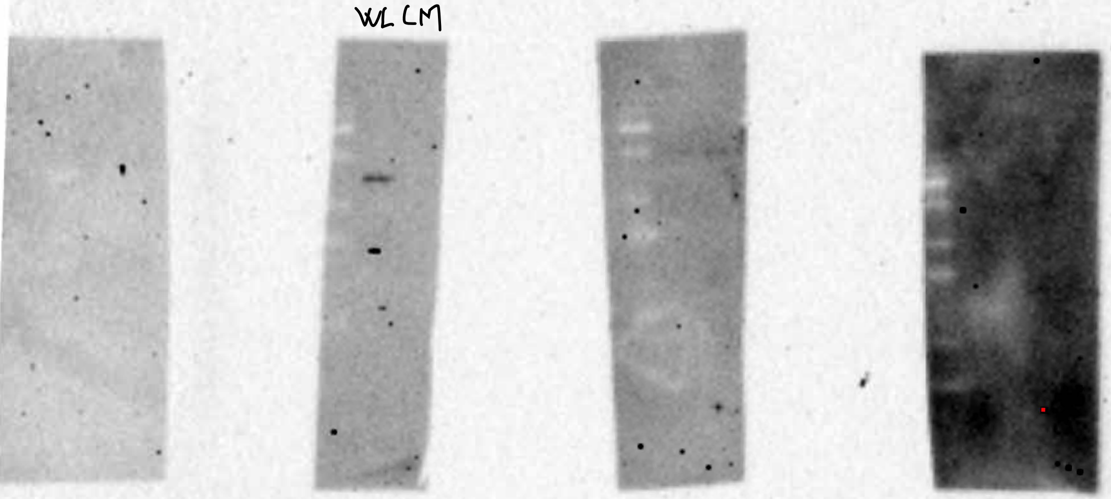

CALNEXIN

Fig. 2A

WL LM

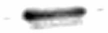

Fig 2A

CD-71

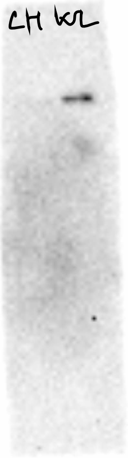

Fig. 2A

Na/K-ATPase

WCLM

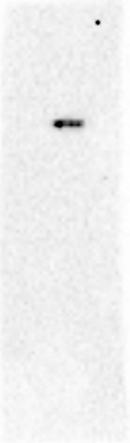

Fig 2A

PHT1

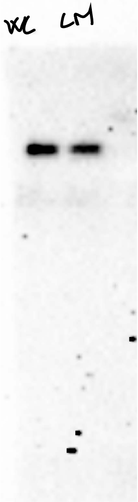

Fig. 2B

PHT1

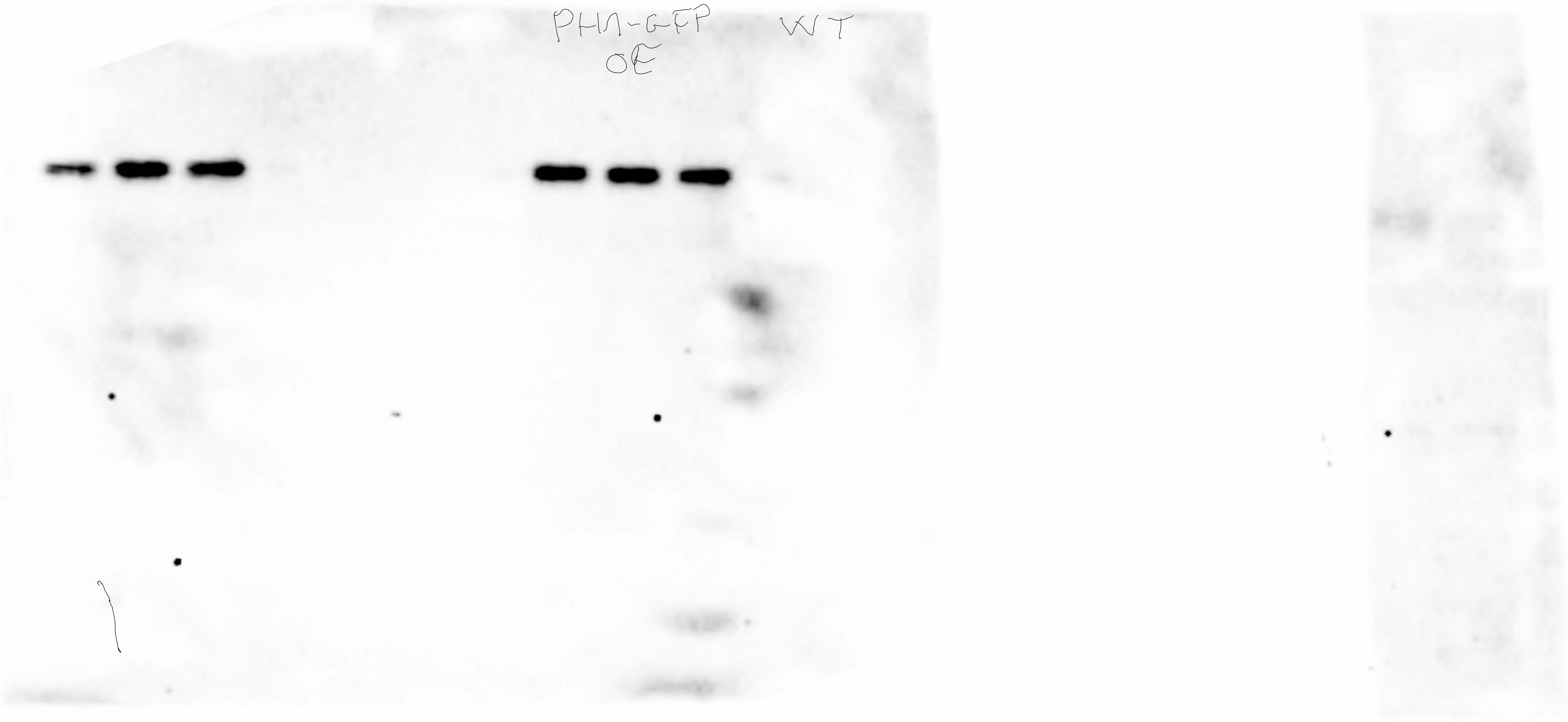

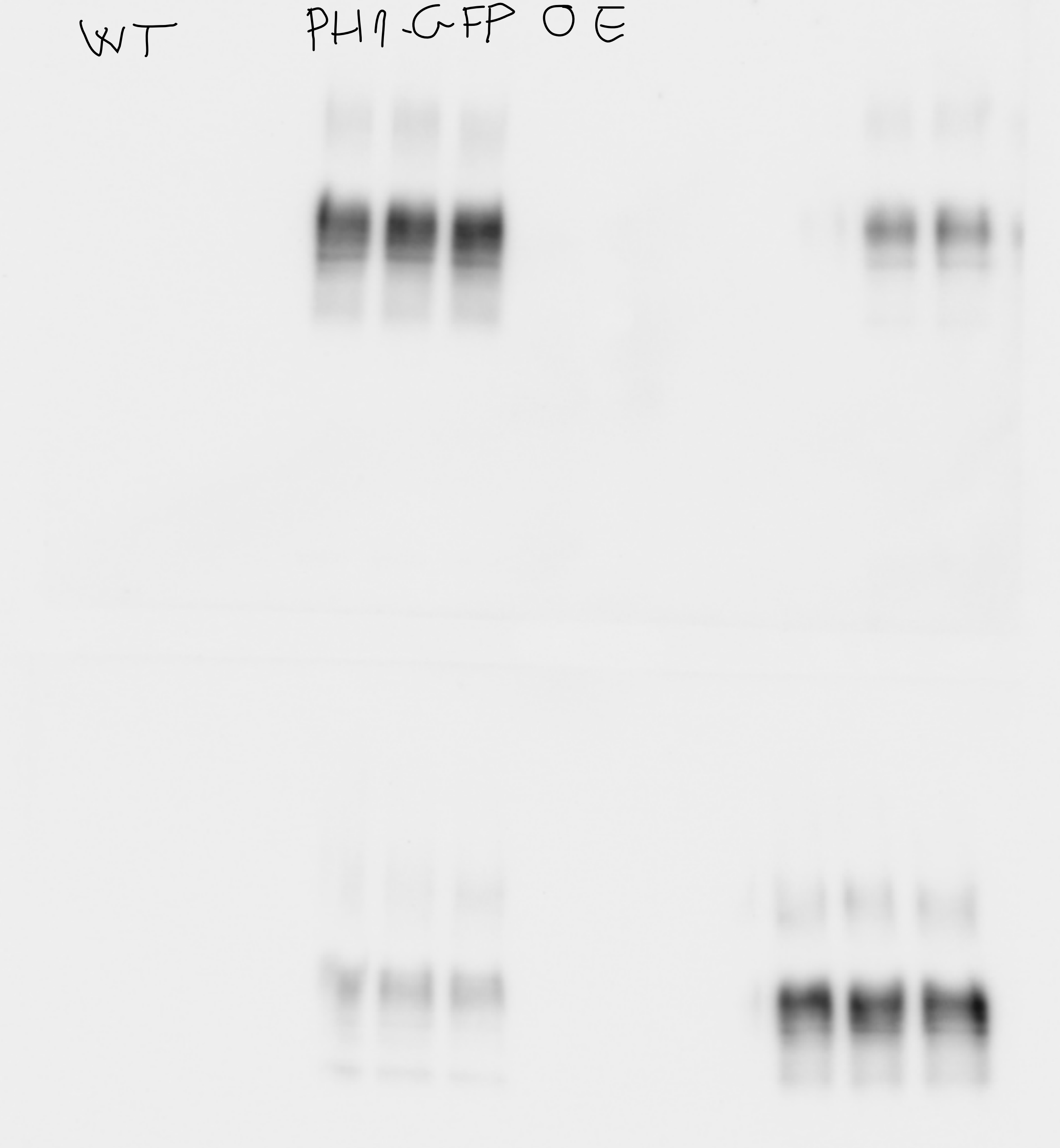

Fig. 2B

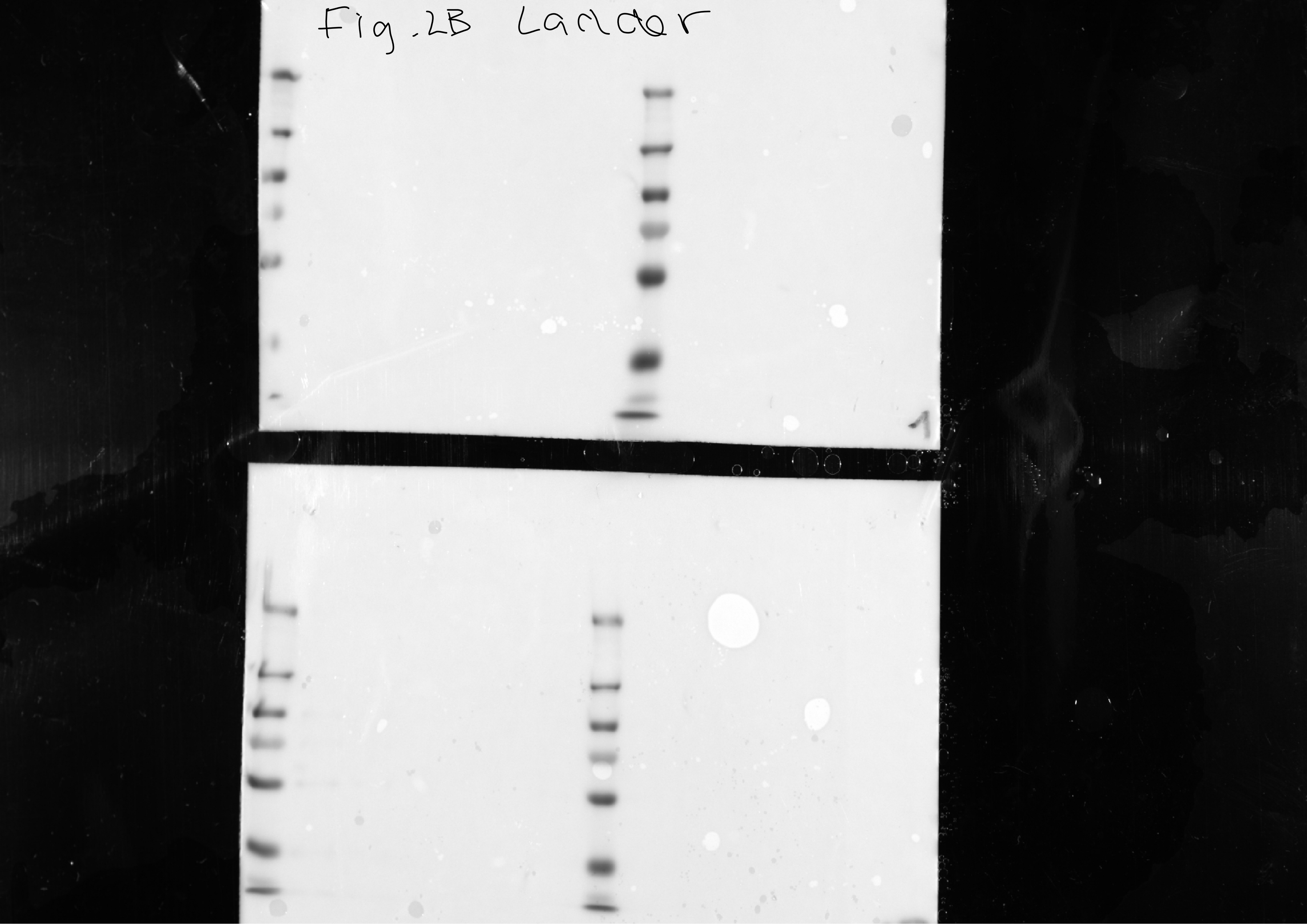
Fig. 2B Ladder
1

Fig. 2B

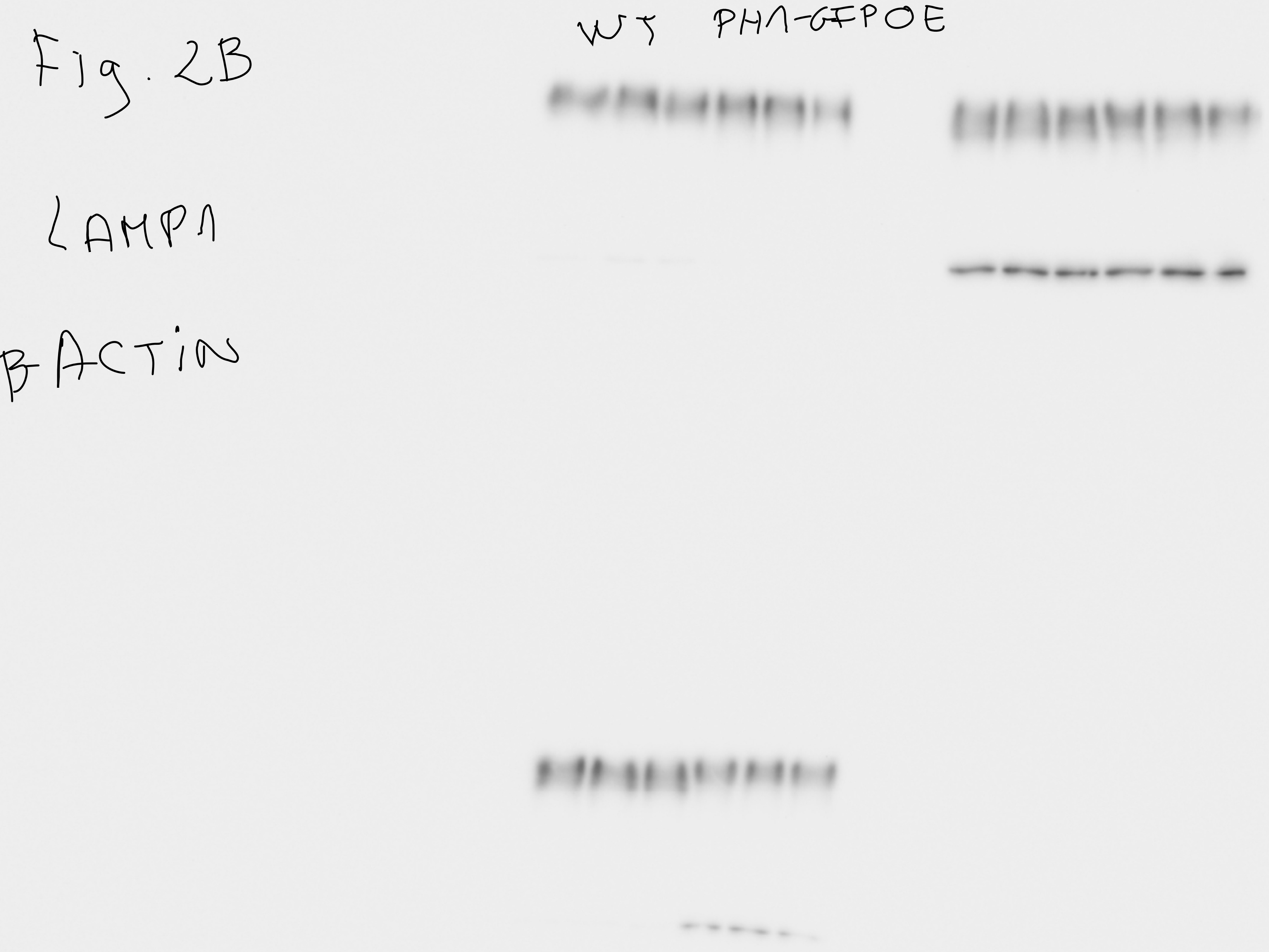

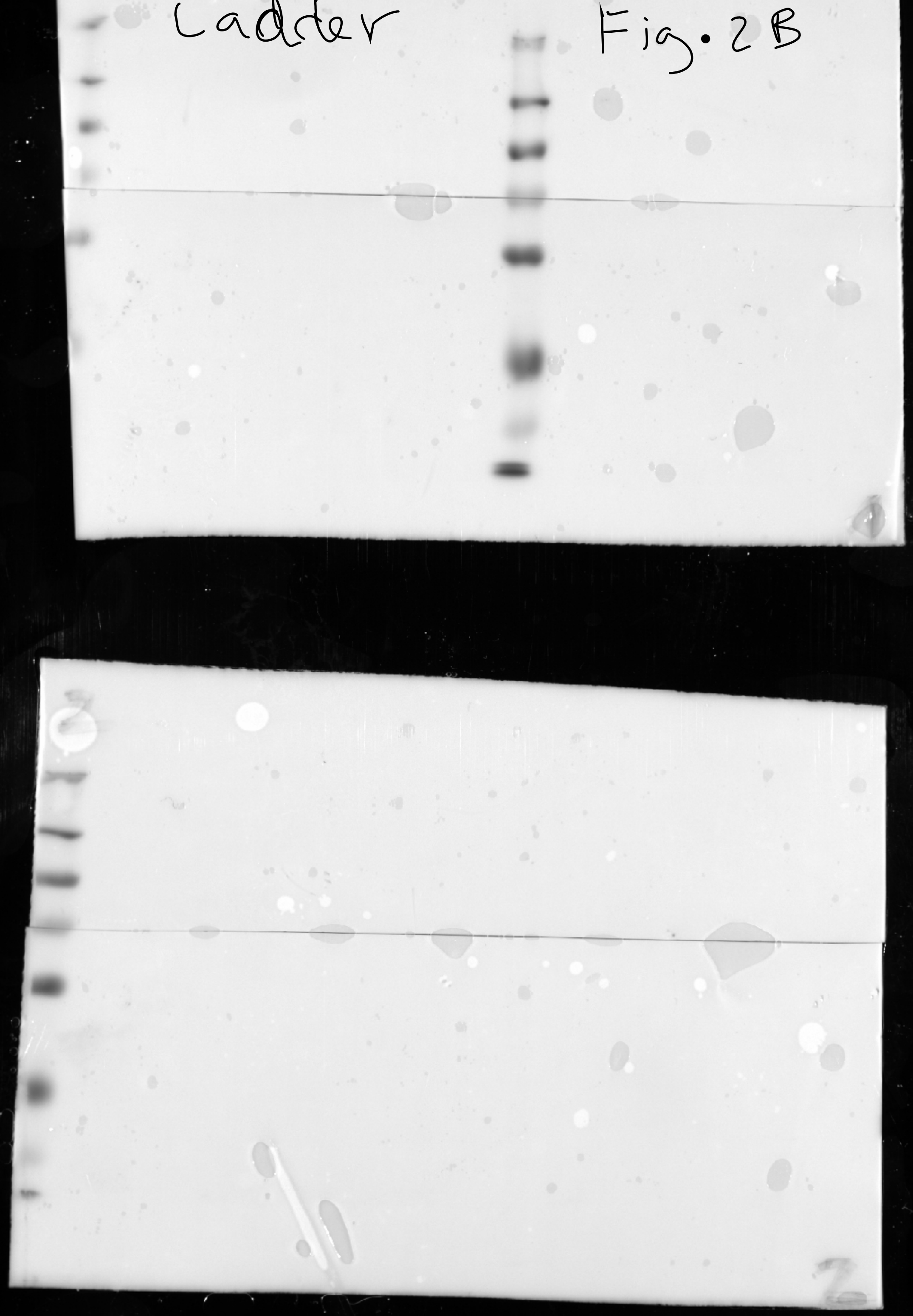

Ladder
Fig. 2B
